# Artificial Intelligence for Precision Oncology of Triple-Negative Breast Cancer: Learning from Melanoma

**DOI:** 10.3390/cancers16040692

**Published:** 2024-02-06

**Authors:** Ornella Garrone, Caterina A. M. La Porta

**Affiliations:** 1 Medical Oncology, Fondazione IRCCS Ca’ Granda Ospedale Maggiore Policlinico, 20122 Milan, Italy; ornella.garrone@policlinico.mi.it; 2 Department of Environmental Science and Policy, University of Milan, 20133 Milan, Italy; 3 Center for Complexity and Biosystems, University of Milan, 20133 Milan, Italy

**Keywords:** triple-negative breast cancer, artificial intelligence, precision oncology

## Abstract

**Simple Summary:**

New technologies, particularly artificial intelligence (AI) and machine learning, enable the utilization of extensive data for personalized medicine. Future challenge involve acquiring limited biological data and extracting information for predictive models. This perspective focuses on triple-negative breast cancer (TNBC), a disease lacking specific treatment, where ongoing investigations explore therapies like immunotherapy. Drawing from the successful use of immunotherapy in melanoma, this perspective explores the identified strengths and weaknesses to inform more successful strategies. In this context, AI and predictive tools are invaluable. Advancements in melanoma immunotherapy serve as a foundation for developing effective immunotherapies for TNBC. Common principles, including immune system activation, checkpoint inhibitors, and personalized treatment, offer prospects for improving the outcomes in aggressive breast cancer cases, presenting new hope for challenging-to-treat patients, avoiding overtreatment, and reducing costs.

**Abstract:**

Thanks to new technologies using artificial intelligence (AI) and machine learning, it is possible to use large amounts of data to try to extract information that can be used for personalized medicine. The great challenge of the future is, on the one hand, to acquire masses of biological data that nowadays are still limited and, on the other hand, to develop innovative strategies to extract information that can then be used for the development of predictive models. From this perspective, we discuss these aspects in the context of triple-negative breast cancer, a tumor where a specific treatment is still lacking and new therapies, such as immunotherapy, are under investigation. Since immunotherapy is already in use for other tumors such as melanoma, we discuss the strengths and weaknesses identified in the use of immunotherapy with melanoma to try to find more successful strategies. It is precisely in this context that AI and predictive tools can be extremely valuable. Therefore, the discoveries and advancements in immunotherapy for melanoma provide a foundation for developing effective immunotherapies for triple-negative breast cancer. Shared principles, such as immune system activation, checkpoint inhibitors, and personalized treatment, can be applied to TNBC to improve patient outcomes and offer new hope for those with aggressive, hard-to-treat breast cancer.

## 1. Introduction

It is well known and established that cancer is highly heterogeneous. This aspect is determined by the great variability between different individuals with the same cancer as well as by the differences between the cells that constitute a specific tumor. Epigenetic intratumor heterogeneity is the basis of changes in the phenotype of tumor cells, but the environment also plays a crucial role. The most important consequence of heterogeneity is the broad response of a specific tumor to therapy or to other environmental stimuli [1,2]. In fact, the high capability of groups of tumor cells to adapt rapidly to another micro-environment by evolving a new cellular ecosystem makes this disease difficult to beat. Phenotypic plasticity refers to the ability of tumor cells to change their behavior and characteristics in response to the changes in their environment. The adaptation leads to important modifications to cell proliferation, migration, and differentiation and to the acquisition of new genetic mutations in a group of tumor cells. Moreover, tumor plasticity can lead to the development of drug resistance. Since the development of secondary tumors or metastases is nowadays the main cause of cancer mortality, it is clear that understanding and addressing tumor plasticity is relevant for the management of oncological patients. In this context, the phenotypic plasticity of tumor cells also plays an important role in immune surveillance too [3].

A critical aspect of tumor plasticity and metastatic capability is the epithelial-mesenchymal-transition (EMT). This is a process by which cells can change from an epithelial, or tightly packed, state to a mesenchymal, or more motile, one. During the EMT, cancer cells lose their cell-cell adhesion properties and gain the ability to migrate and invade. The EMT also confers resistance to cell death, making these cells more likely to survive and form new tumors.

The EMT is a complex process that is regulated by a variety of signaling pathways, including those activated by growth factors and cytokines. Recent studies have identified several key transcription factors and signaling pathways that are responsible for the induction of the EMT, including the Snail, Twist, and Zeb families of transcription factors and the TGF-beta and WNT signaling pathways. The EMT is also a reversible process, and mesenchymal cells can also revert to the epithelial state by undergoing mesenchymal–epithelial transition (MET). Recent evidence clearly shows that when epithelial cancer cells acquire a mesenchymal gene program, they increase their capability for migration and invasion [4,5]. Moreover, considerable evidence illustrates the importance of intermediate states where cells express markers of both E and M states, with these showing higher aggressiveness than cells in the M state [6].

To obtain a pictorial view of phenotypic plasticity, our group used Boolean network models of the EMT to represent the phenotypes as epithelial (E) cells transformed into mesenchymal (M) cells [6]. By analyzing all the possible phenotypic states, we were able to reconstruct the topography of the phenotypic landscape, a concept initially conceived in broad terms by Waddington several decades ago [7]. Notably, our exploration revealed a multitude of intermediate E/M states, challenging the conventional, rigid distinction between these cell types. This discovery underscores the need to move away from categorical definitions, impacting the utility of biomarkers for defining hybrid cellular states due to the existence of a continuum of potential phenotypes with varying degrees of plasticity [6,8]. In light of these insights, the existence of dormant cells can be interpreted as merely one state within a complex relationship with the environment [5,8].

Another critical aspect of metastasis is the capability of tumors cells to detach from the tumor mass, changing their states from a collective or flowing state into a jamming one characterized by limited mobility [9]. Jamming-unjamming transitions (JUT) and their reverse the unjamming-jamming transitions (UJT), were traditionally observed in the rheology of soft materials [10] but are now becoming widely discussed in cell biophysics. An interesting debate revolves around the possible connection between EMT/METs and JUT/UJTs. It is not clear if the two types of transitions are related, acting together, or if they are they mutually exclusive. These aspects are particularly intriguing, as the transition to a coupled unjammed, or active nematics, state appears sometimes in collective migration, when cells can move while remaining attached [11]. This state bears resemblance to the hybrid Epithelial/Mesenchymal (E/M) state, and it remains uncertain whether hybrid E/M states are essential for collective cell migration. Our research group recently addressed this question by employing the principles of non-equilibrium phase transitions and critical phenomena [11]. In essence, we propose that EMT/MET and UJT/JUT represent two fundamentally different types of transitions [11]. Cells undergoing an EMT can induce a JUT in the assembly by reducing cell-cell adhesion, a crucial parameter driving the JUT. Conversely, cells transitioning to a jammed state non-necessarily increase their mutual adhesion through an MET, a phenomenon previously referred to as the adhesion paradox [9].

## 2. Cellular Plasticity and New Therapeutic Strategies in Other Tumors: The Example of Melanoma

Drug resistance is a critical barrier for the treatment of many tumors including triple-negative breast cancer (TNBC), since it implies the possibility of having dormant cancer cells in the presence of a permissive environment. However, the treatment of tumors with specific drugs might help the selection of dormant cells or help cells to become senescent and, therefore, contribute to keeping the cells viable for an extended period of time. Resistance to drugs poses a significant barrier to effectively treating cancer plasticity, as existing medications often target general biological aspects of tumors without considering individual tumor specificity. Exploring the lessons learned from immunotherapy experiences in diverse cancers, such as melanoma, provides valuable insights applicable to triple-negative breast cancer (TNBC), given their shared high heterogeneity. Over the past decade, immunotherapies have transformed cancer treatment, with immune checkpoint blockade (ICBs) against programmed cell death protein 1/programmed cell death ligand 1 (PD-1/PD-L1) and cytotoxic T-lymphocyte antigen-4 (CTLA-4) axes emerging as the frontline therapy for metastatic melanoma. Additionally, adoptive T-cell transfer therapy is actively being examined in preclinical melanoma models, aiming to enhance cytotoxic T-cell functions for improved anti-tumor immune responses [12,13,14]. However, challenges include innate anti-PD-1 resistance gene signatures (IPRES) identified in melanoma samples from non-responders to anti-PD-1 immunotherapy [15]. Melanoma can develop acquired resistance through phenotype switching, with invasive phenotypes associated with a heightened myeloid-derived suppressor cell (MDSC) infiltration and WNT5T secretion, driving an invasive state [16]. Conversely, phenotype switching plays a prognostic role in predicting responders, with a high mutation rate and the appearance of neoantigens improving responses to anti-PD-1 [16].

The possibility of interfering with phenotypic switching in melanoma seems to be, therefore, an interesting way to overcome resistance and the spread of tumor cells. To better understand the molecular mechanisms underlying this process, a recent paper showed how a complex network of miRNAs, including miRNA222, drives melanoma plasticity through the EMT transition, [17,18]. Moreover, communication between the different subpopulations of tumor cells such as cancer stem cells (CSCs) and cancer cells (CSs) mediated by miRNAs (i.e., miRNA222), was shown to drive the EMT transition, balancing the ratio between CSCs and CS cells [17]. Accordingly, the KO of miRNA222 was able to inhibit the EMT transition and the plasticity of the tumor cells [18].

In order to deeply understand the development of heterogeneity in melanoma, our group has also recently investigated the biophysical changes occurring during phenotypic switching [19]. This study highlights two interesting aspects that should be further investigated: (1) cellular heterogeneity involves crucial biophysical nuclear changes, i.e., blebbiness and stiffness, and (2) remodelling of polycomb-chromatin/lamin/cytoskeleton occurs during phenotypic switching [19].

## 3. Mechanisms of Immune Evasion and Resistance: What We Can Learn from Melanoma

Melanoma is known for its ability to evade the immune system, which is thought to play a key role in the development and progression of the disease. Some of the mechanisms of immune evasion and resistance in melanoma include: (a) downregulation of immune cell recognition: melanoma cells can downregulate the expression of molecules on their surface that are recognized by immune cells, making it more difficult for the immune system to detect and attack them; (b) inhibition of immune cell activation: melanoma cells can secrete factors that inhibit the activation of immune cells, preventing them from mounting an effective response against the cancer; (c) induction of immune cell apoptosis. Melanoma cells can induce the apoptosis (programmed cell death) of immune cells, further weakening the immune response; (d) development of a tumor microenvironment (TME) that promotes immune tolerance: melanoma cells can secrete factors that recruit immune cells that suppress the immune response, creating a TME that is tolerant to the cancer; (e) mutation of key immune genes: Melanoma cells can mutate genes that are important for the immune response, making it more difficult for the immune system to recognize and attack them; (f) epigenetic changes that alter gene expression. Melanoma cells can undergo epigenetic changes that alter gene expression, including genes that are involved in the immune response, allowing them to evade the immune system.

These mechanisms of immune evasion and resistance in melanoma are complex and interrelated, and understanding them is critical for developing effective treatments for the disease. Researchers are currently investigating a variety of immunotherapies, including checkpoint inhibitors and adoptive cell therapies, to overcome these immune evasion mechanisms and improve outcomes.

The immune checkpoint blockade constitutes a form of cancer immunotherapy targeting immune checkpoints pathways, which are very important for the immune response. By inhibiting these pathways, the immune system is driven into a more potent and effective response against cancer. The two most well-known immune checkpoints are CTLA-4 and PD-1, in addition to its ligand PD-L1. These immune checkpoints play a critical role in regulating T cell activity, which are the immune cells responsible for recognizing and attacking cancer cells [20]. CTLA-4 blockade therapy was the first ICB approved by the FDA for the treatment of metastatic melanoma in 2011. By blocking CTLA-4, this therapy enhances T cell activation and proliferation, leading to a more robust immune response against cancer cells. PD-1/PD-L1 blockade therapy, on the other hand, blocks the interaction between PD-1 on T cells and PD-L1 on cancer cells [20]. This interaction inhibits T cell activation and leads to T cell exhaustion, which is a state of reduced T cell activity that allows cancer cells to evade immune detection. By blocking this interaction, PD-1/PD-L1 blockade therapy restores T cell activity and promotes an immune response against cancer cells [20].

Compared to conventional therapies, immunotherapy seems to induce durable responses in patients with metastatic cancers. However, there are advantages and limitations. While surgery is potentially curative, radiation therapy and chemotherapy often have limitations, including toxicity, lack of specificity, and the potential for cancer cells to develop resistance over time. In contrast, ICB therapy is a type of immunotherapy that harnesses the power of the immune system to recognize and attack cancer cells. By targeting immune checkpoints that are overexpressed in melanoma cells, these therapies can enhance the immune response against the cancer and lead to durable responses.

Therefore, one major advantage of ICBs over conventional therapy is that they can result in long-lasting responses, with some patients experiencing complete remission and remaining cancer-free for years. In contrast, conventional therapies often have limited efficacy and may require frequent retreatment or the use of multiple therapies in combination to achieve a response.

Another advantage of an immune checkpoint blockade is its specificity, as it targets cancer cells specifically while sparing healthy cells. This can reduce the risk of side effects associated with conventional therapies, such as hair loss, nausea, and damage to healthy tissues.

However, immune checkpoint blockade therapy is not without limitations. While some patients respond well to these therapies, one-third of patients responding to immunotherapy in most cancer types experience serious autoimmune side effects [13]. Additionally, these therapies are often expensive and require specialized training and expertise to administer and manage.

Overall, ICB therapy offers a promising new approach to the treatment of melanoma and other cancers, but it should be used in conjunction with conventional therapies and tailored to the individual patient’s needs and medical history. Moreover, combinations of immunotherapies have shown improved outcomes but also result in more severe side effects than single-agent therapy [21].

Since not all patients respond to immunotherapy and some may develop resistance over time, the effectiveness of immunotherapy in melanoma can be influenced by several factors, including: (a) The tumor mutational burden (TMB): the TMB is the number of genetic mutations present in a tumor. Melanoma often has a high TMB, which can increase the likelihood of producing neoantigens, or abnormal proteins that the immune system can recognize as foreign and attack. Patients with a high TMB may be more responsive to immunotherapy. (b) Tumor-infiltrating lymphocytes (TILs): TILs are immune cells that infiltrate the TME. Patients with high levels of TILs may be more responsive to immunotherapy. (c) PD-L1 expression: PD-L1 is a protein that is often upregulated in cancer cells and can interact with immune cells to inhibit their activity. Patients with high PD-L1 expression may be more responsive to PD-1 or PD-L1 checkpoint inhibitors. (d) The presence of other immune cells: the presence of other immune cells in the TME, such as regulatory T cells (Tregs) or myeloid-derived suppressor cells (MDSCs), can inhibit the activity of immune cells and reduce the effectiveness of immunotherapy. (e) The timing of treatment: the timing of immunotherapy treatment can also impact its effectiveness. For example, neoadjuvant immunotherapy given before surgery may be more effective than adjuvant immunotherapy given after surgery. (f) Patient characteristics: factors such as age, sex, and overall health can also influence the effectiveness of immunotherapy. Recently, the possible impact of the microbiome on the effectiveness of immunotherapy was proposed.

The microbiome, which is the collection of microorganisms that inhabit the human body, has been shown to play an important role in modulating the immune system and may influence the effectiveness of immunotherapy in melanoma.

Several studies have suggested that the composition of the gut microbiome in particular can impact the efficacy of immunotherapy in melanoma. For example, patients with melanoma who have a higher diversity of gut bacteria have been shown to have better responses to anti-PD-1 therapy [22]. One proposed mechanism for this effect is that certain bacteria in the gut microbiome can stimulate the immune system and promote the activity of T cells, which are important immune cells that can attack cancer cells. Additionally, some bacteria may produce metabolites that can enhance the activity of immune cells or alter the tumor microenvironment to make it more favorable for immune cell activity. However, the exact mechanisms by which the gut microbiome influences the effectiveness of immunotherapy in melanoma are still not fully understood, and further research is needed to determine the specific bacterial strains and metabolites that are involved.

Overall, the microbiome may represent a promising target for improving the effectiveness of immunotherapy in melanoma, and ongoing studies are investigating the potential of using probiotics, prebiotics, or fecal microbiota transplantation (FMT) to modulate the gut microbiome and enhance the efficacy of immunotherapy [23].

In summary, immune checkpoint blockade therapy has shown promising results in the treatment of several types of cancer, including melanoma. However, not all patients respond to these therapies, and some experience side effects such as autoimmune reactions. Ongoing research is focused on improving patient selection, identifying new biomarkers that can predict responses, and developing combination therapies that can enhance the efficacy of immune checkpoint blockades. For all these reasons, the use of new strategies using artificial intelligence (AI) that can help to identify the subset of patients where these strategies will be more successful or that can follow the patient response during the treatment is the next step of ongoing research in the field of cancer immunotherapy. The most significant improvement might be: (1) Patient selection; (2) Developing new biomarkers: while current biomarkers like the PD-L1 expression are helpful in predicting responses, there is still a need for additional biomarkers that can better predict which patients are most likely to benefit from immune checkpoint blockades; (3) Combination therapies: combining immune checkpoint blockades with other treatments, such as chemotherapy or targeted therapies, may enhance the efficacy of these therapies in certain patients; (4) Optimization of the treatment regimens for individual patients taking into account factors such as the TME and patient characteristics.

Overall, we do believe that the possibility of better stratifying patients and following them to determine the real impact of immunotherapy on tumor progression has two main overall objectives: (1) to reduce unnecessary treatment of patients and (2) to reduce costs, which in particular for immunotherapy are high.

## 4. What We Can Learn form Melanoma for Treating TNBC Successfully

Melanoma and triple-negative breast cancer (TNBC) share several significant similarities. Both are characterized by their aggressiveness and resistance to standard therapies, making them challenging to treat [24]. They also exhibit higher tumor mutational burdens, meaning they have more genetic mutations compared to other cancer types, which can make them more responsive to immunotherapies that the target unique genetic features of the tumor [25]. Additionally, both melanoma and TNBC often involve the evasion of the immune system, and research in melanoma has shed light on strategies to activate the immune response, such as checkpoint inhibitors, which are now being explored in TNBC treatment. Understanding these commonalities between melanoma and TNBC can inform the development of innovative, targeted treatment approaches that harness the power of immunotherapy to combat these aggressive cancers.

## 5. Triple Negative Breast Cancer: The Old and the New Therapeutic Strategies

TNBC is a subtype of breast cancer characterized by certain distinctive features, briefly summarized in the following [26]: (1) TNBC does not express the three specific receptors commonly found in breast cancer cells: estrogen receptors (ERs), progesterone receptors (PRs), and human epidermal growth factor receptor 2 (HER2/neu). This lack of receptor expression makes TNBC distinct from other breast cancer subtypes that may be treated with targeted therapies directed at these receptors. (2) TNBC tends to be more aggressive and fast-growing compared to other types of breast cancer. It often has a higher rate of cell proliferation and may be associated with a higher risk of early recurrence. (3) Since TNBC lacks estrogen, progesterone, and HER2 receptors, it does not respond to hormone therapy (e.g., tamoxifen or aromatase inhibitors) or HER2-targeted therapies (e.g., trastuzumab). As a result, treatment options for TNBC are more limited, primarily involving chemotherapy. TNBC is more likely to occur in younger women, particularly those under the age of 50. There is also a higher prevalence of TNBC among individuals with BRCA1 gene mutations and in certain ethnic groups, such as African Americans. Moreover, TNBC is highly heterogeneous and has a higher risk of metastasis [27]. This can make treatment and management more challenging, and, in general, TNBC is associated with a less favorable prognosis compared to other breast cancer subtypes.

The treatment of TNBC typically involves a combination of conventional therapies and, in some cases, emerging immunotherapy options. The specific treatment plan for an individual with TNBC depends on factors like the stage of cancer, the extent of spread, the patient’s overall health, and more. Surgery is often the first step in treating TNBC, and after surgery, radiation therapy may be recommended to target any remaining cancer cells and reduce the risk of local recurrence. Chemotherapy is a standard treatment for TNBC, and it is often administered before or after surgery (neoadjuvant or adjuvant chemotherapy, respectively) or in cases of advanced or metastatic disease. The most used chemotherapy drugs are anthracyclines, taxanes, and carboplatin. The choice of chemotherapy regimen may vary based on the individual case and the response to treatment. Immunotherapy has emerged as a promising treatment option in PD-L1-positive metastatic TNBC patients. The results of the Impassion 130 and KEYNOTE-355 study led to the approval of both anti PD-L1 atezolizumab and anti PD-1 pembrolizumab for the treatment of PD-L1-positive metastatic breast cancer [28,29,30]. Recently, Pembrolizumab has been approved also for the treatment of patients with operable breast cancer in addition to chemotherapy. The results of the KEYNOTE-522 study demonstrated an increased percentage of pathological complete responses and a significant prolongation of event-free survival after the addition of pembrolizumab to a backbone chemotherapy regardless of PD-L1 status [28].

Targeted therapy of TNBC also involves the use of drugs that specifically target certain molecules or pathways involved in the growth and spread of cancer cells. Unlike hormone therapy or HER2-targeted therapy, which are not effective in TNBC due to the lack of hormone receptors and HER2 expression, targeted therapies are designed to exploit other vulnerabilities in the cancer cells. In summary, there are PARP (Poly-ADP ribose polymerase) inhibitors, which are a type of targeted therapy used in TNBC patients with BRCA1 or BRCA2 gene mutations. These mutations disrupt DNA repair mechanisms in cancer cells, making them more reliant on the PARP pathway for repair. PARP inhibitors, such as olaparib and talazoparib, block this repair mechanism, leading to DNA damage accumulation and cancer cell death. This targeted therapy is particularly effective in BRCA-mutated TNBC cases [31]. Some TNBC tumors overexpress the epidermal growth factor receptor (EGFR). EGFR inhibitors like cetuximab and erlotinib can be used in select cases where EGFR expression is high [31]. These drugs work by blocking the EGFR signaling pathway, which is involved in cell growth and division. The mTOR (mammalian target of rapamycin) pathway plays a role in cell growth and proliferation. mTOR inhibitors like everolimus have been investigated in clinical trials for TNBC, either alone or in combination with chemotherapy or other targeted therapies [31]. It is important to note that not all TNBC patients will benefit from targeted therapies, as these treatments are typically effective in subsets of TNBC cases with specific molecular characteristics. Finally, recently, antibodies drug conjugates (ADCs) have been approved for the treatment of metastatic TNBC [32].

## 6. Systems Biology and AI in Cancer Research: A Brief Overview

Systems biology is an interdisciplinary field of study that aims to understand the complex interactions within biological systems by considering them to be integrated and interconnected networks. Unlike traditional reductionist approaches that focus on individual components, such as genes or proteins, systems biology takes a holistic perspective, emphasizing the dynamic relationships and emergent properties that arise from the collective behavior of these components.

In systems biology, living organisms are viewed as complex systems with multiple levels of organization, from molecules and cells to tissues and entire organisms. This field integrates principles from biology, mathematics, computer science, physics, and engineering to develop computational models and simulations that capture the behavior of biological systems. By employing quantitative and computational methods, in systems biology, we seek to unravel the underlying principles governing biological processes and their regulation through the construction and analysis of biological networks, such as gene-regulatory networks, signaling pathways, and metabolic pathways. Mathematical models, including differential equations and stochastic models, are used to describe and simulate the behavior of biological systems, with the goal of predicting system responses to perturbations and providing insights into the dynamics of biological processes. The integration of data from diverse datasets, including genomics, transcriptomics, proteomics, and metabolomics, is performed to capture comprehensive molecular profiles of a biological system, enabling a deeper understanding of complex biological phenomena. Systems biology often leverages AI in several ways to enhance its capabilities and address the complexity of biological systems. Several achievements in cancer research have stemmed from the combined use of systems biology and AI, particularly in the discovery of new drugs [33,34] as well as in the search for novel biomarkers critical for early detection and accurate diagnosis [35,36,37].

## 7. Precision Oncology and Possible Use of Artificial Intelligence: The Developments in Melanoma

Melanoma is a strongly heterogeneous cancer, ruled by the interplay of several genetic and environmental factors, and it is extremely challenging to treat its advanced stages. Clinical decisions are complicated to make because of the complexities deriving from the development of intrinsic or secondary resistance which often lead to therapeutic failures. Within a 5-year time frame, around 70% of the patients face disease progression encompassing both primary resistance, observed in 30–50% of patients, and secondary resistance, affecting an additional 20–30% of patients experiencing relapse despite initial treatment benefit [38]. Primary resistance to a single agent is defined in patients who have received at least 6 weeks of therapy and is assessed considering the development of either confirmed radiographic progression, as evidenced by two imaging tests conducted at least 4 weeks apart, or unequivocal clinical progression within 6 months of initiating treatment and while actively on therapy. Secondary resistance, on the other hand, is defined in patients that have received therapy for a minimum of 6 months and who initially obtained clinical benefit. This is defined as a complete or partial response or stable disease for at least 6 months. Secondary resistance is assessed similarly to primary resistance: it requires confirmed radiographic progression with two imaging tests spaced at least 4 weeks apart or unequivocal clinical progression. The classification of resistance post-therapy discontinuation, whether it occurs after the completion of adjuvant/neoadjuvant therapy, after attainment of the maximal benefit, or due to severe toxicity, corresponds to either primary or secondary resistance. The differentiation between primary and secondary resistance depends on the initial response and the time passed since the last treatment, with a commonly agreed-upon cutoff of 12 weeks [39].

Adjuvant immunotherapy has brought about a significant transformation in the therapeutic approach for those dealing with advanced melanoma, representing a pivotal shift in the treatment landscape. Despite this noteworthy progress, the domain of adjuvant treatment presents unique challenges, emphasizing the need to identify biomarkers for discerning individuals who would benefit from such interventions. The determination of the optimal duration and intensity of treatment also introduces complexities in adjuvant therapy. Furthermore, the management of recurrence following adjuvant immunotherapy has gained increasing importance, given the growing utilization of these adjuvant approaches, leading to a rising number of patients experiencing relapses. Additionally, the substantial toxicity linked with these treatments, alongside the economic implications, further underscores the multifaceted considerations within the adjuvant immunotherapy domain.

Although there is still substantial work to be undertaken, a wealth of evidence indicates that harnessing artificial intelligence (AI) technologies capable of processing extensive datasets holds considerable promise for improving the care of patients with advanced melanoma. These AI applications have the potential to assist clinicians in pinpointing the most advantageous therapeutic options, thereby avoiding unnecessary and costly treatments that may result in adverse side effects. This strategic approach is in line with the principles of precision oncology, emphasizing the importance of tailoring treatments according to individual patient characteristics.

The demand for precision oncology underscores the need for robust predictive tools, especially within the realm of immunotherapy. While immunotherapy has proven to be highly effective in specific cases, its significant cost implications pose challenges. Therefore, it is crucial to proactively identify the patients most likely to respond to immunotherapy, optimizing treatment outcomes and resource allocation.

For these purposes, there are some areas where AI can contribute significantly and appear promising from our point of view:

(1) Radiomics has the transformative capability to convert radiological images into quantitative data, facilitating the extraction of crucial biological information. The utilization of artificial intelligence (AI) to explore extensive image datasets has proven instrumental in the development of diagnostic and prognostic models. In the specific context of melanoma, studies have delved into therapy management by identifying predictive biomarkers through radiological analyses [40]. For instance, a recent investigation assessed the predictive role of the radiomic analysis of magnetic resonance images (MRIs) in response to immunotherapy for melanoma brain metastases [41]. Complementarily, the integration of radiomics with biological markers has yielded intriguing results in melanoma patients undergoing immunotherapy [41]. Another study highlighted the utility of AI in radiomics for lung cancer, providing a valuable tool for risk prediction, diagnosis, and prognosis [42].

Furthermore, the convergence of radiogenomics and AI has emerged as a potent synergy propelling personalized medicine forward. This interdisciplinary approach harmonizes radiological imaging with genomic data, unraveling intricate relationships between a patient’s unique genetic makeup and the radiographic characteristics of tumors [43]. AI algorithms, with their adeptness in discerning subtle patterns within extensive datasets, play a pivotal role in decoding complex radiogenomic information. By amalgamating genomic and radiological data, clinicians can gain a deeper understanding of the molecular underpinnings of a patient’s disease, facilitating more precise and tailored treatment strategies. AI-driven analyses contribute to the identification of radiomic signatures correlated with specific genetic mutations, enhancing the ability to predict disease behavior and treatment responses. This fusion of radiogenomics and AI not only elevates the diagnostic accuracy but also lays the groundwork for personalized therapeutic interventions, ultimately optimizing patient outcomes in the era of precision medicine [43].

(2) New tools using AI for disentangle cancer heterogeneity. In this context, ARIADNE is a recently described and validated algorithm for triple-negative breast cancer that is able to stratify the aggressiveness of the tumor in patients based on their complex gene expression data [6]. The capability to scale up ARIADNE to others tumors as well as the potential and limit of AI in this field is described and discussed in a recent paper [44];

(3) Leveraging artificial intelligence (AI) in analyses of extensive datasets to discern the optimal therapeutic course. In the context of melanoma, such strategies hold promise for predicting potential disease recurrence and anticipating responses to standard treatments, thereby facilitating the exploration of diverse treatment scenarios [39];

(4) AI and machine learning can be useful to reposition drugs that were unsuccessful, as discussed in these recent reviews [45,46].

## 8. Algorithmic Methods for TNBC

In the last decade, there has been a notable surge in the development of gene expression tests tailored specifically for triple-negative breastcCancer (TNBC) [47,48,49]. Lehmann et al. [47], proposed a data-driven classification system for TNBC, initially suggesting four subtypes characterized by distinct gene expression patterns and unique biological features. Further analysis led to a new classification with six subtypes [50]: basal-like 1, basal-like 2, immunomodulatory, mesenchymal, mesenchymal stem-like, and luminal androgen receptor (LAR) subtypes. The basal-like subtypes, marked by a heightened expression of basal markers, are recognized for their increased aggressiveness, while the immunomodulatory and LAR subtypes are considered less aggressive, associated with more favorable outcomes. The mesenchymal and mesenchymal stem-like subtypes are defined by a heightened invasiveness and metastatic potential. Despite evidence suggesting differential responses to therapy among these six TNBC subtypes [50], no statistically significant differences in relapse-free survival have been established so far [50].

A more recent alternative is the ARIADNE algorithm, which was developed by one us. ARIADNE is a general framework to study cancer cell plasticity based on Boolean network model simulations of the pathway controlling the EMT [6]. The model is used to construct a landscape of the possible cell phenotype on which we can map gene expression data [51]. It has been recently shown that ARIADNE is able to predict the aggressiveness of TNBC from gene expression data [51]. Comparing the ARIADNE score with gene expression signatures based on the tumor immune microenvironment, it was also found that ARIADNE is able to identify a high-risk TNBC population with high immune markers that is, however, not properly classified by the tumor immune microenvironment-based strategy [52]. In a recent paper [53], ARIADNE was used to study the response to chemotherapy (anthracycline/taxane) of triple-negative breast cancer patients. The results showed that the ARIADNE score is correlated with pCR rates, and that within the group of patients responding to treatment, ARIADNE is associated with disease-free survival.

## 9. Conclusions and Future Directions

New technologies such as AI and machine leaning are opening up new avenues in the management of cancer patients from diagnosis to prognosis, and above all, the possibility of creating new predictive tools appears to be very innovative and will be expanding in the near future. The great advantage of these new technologies is the possibility of avoiding unnecessary treatments for the patient, being more timely and also being able to monitor the patient over time. Also, from an economic point of view, these strategies allow for a move towards more sustainable treatments, considering in particular the high cost of immunological therapies, in addition to their high toxicity. In the future, we must increasingly aim for prevention in healthy subjects before they develop the disease using non-invasive predictive tools. ARIADNE allows us to calculate aggressiveness in patients with TNBC, assisting the further management of these patients, and it is the first platform that allows for personalized oncology.

It is also necessary from our point of view to have more scientists in the near future who have a multidisciplinary background or who work in multidisciplinary teams. It is becoming increasingly necessary to use complementary skills to deal with complex problems.

Moreover, discoveries in immunotherapy for melanoma hold significant promise for the future treatment of triple-negative breast cancer (TNBC) with immunotherapy due to several key factors and principles shared between these two types of cancer: (1) Immune System Activation: Both melanoma and TNBC are known for their ability to evade the immune system, allowing the cancer to grow and spread. Immunotherapy in melanoma has provided insights into ways to activate the immune system effectively. This can be translated to TNBC treatment, as it may involve similar mechanisms to boost the immune system’s response against the tumor. (2) Checkpoint inhibitors: Immunotherapy for melanoma has introduced the use of checkpoint inhibitors, which are drugs that block proteins like PD-1 and CTLA-4 to prevent cancer cells from evading immune system detection. Checkpoint inhibitors have shown promise in melanoma and are now being investigated for their effectiveness in TNBC treatment. Early studies suggest that checkpoint inhibitors may have a positive impact on TNBC by restoring the immune system’s ability to recognize and attack cancer cells. (3) The Tumor Mutational Burden (TMB): Both melanoma and TNBC tend to have a higher tumor mutational burden, which means they have more genetic mutations compared to other cancers. A high TMB is often associated with a better response to immunotherapy, as the immune system can target the unique genetic features of the tumor. Insights from melanoma research regarding the TMB and immunotherapy responses can inform TNBC treatment strategies. (4) The Tumor Microenvironment: The tumor microenvironment, which includes immune cells, blood vessels, and other components, plays a crucial role in cancer development and response to treatment. Understanding how the tumor microenvironment changes in response to immunotherapy in melanoma can guide researchers in tailoring similar approaches for TNBC. Strategies that reshape the tumor microenvironment to promote immune attack can be applied across different cancer types. (5) The presence of tumor-infiltrating lymphocytes (TILs), which consist of all lymphocytic cell populations that have invaded the tumor tissue, can favour the treatment with ICT. (6) Combination Therapies: Melanoma research has shown the potential benefits of combining different immunotherapy approaches, such as checkpoint inhibitors with targeted therapies or other immune-boosting agents. These combination therapies could be adapted for TNBC treatment to enhance the effectiveness of immunotherapy by addressing multiple aspects of tumor evasion and resistance. (7) Personalized Medicine: Advances in melanoma immunotherapy have led to a greater emphasis on personalized medicine. Tailoring treatment approaches to the unique genetic and immunological characteristics of each patient’s cancer is a promising strategy. This approach can be applied to TNBC, allowing for more precise and effective treatment regimens.

Another new aspect that could produce interesting developments, but is currently in its early stages, is spatial molecular imaging for the examination of the spatial landscapes and transcriptional profiles of complex tissues at a subcellular resolution [54,55]. This kind of approach might contribute to uncovering the complex spatial architecture within heterogeneous tissues and therefore facilitate the acquisition of biological insights. If this type of approach were to become routinely used and therefore allow the collection of images, the use of AI could help to untangle the complex heterogeneity of tumors with a view to personalized therapy. Furthermore, with the possibility of carrying out investigations using this kind of approach, the resistant tumor cells interacting with themselves or the surrounding constituents to form an ecosystem for drug resistance seems to be an interesting perspective due to the reported heterogeneous sensitivity or resistance to drugs of the tumor cells in different locations of a tumor lesion [55].

## Data Availability

No data were generated for this paper.
